# Prognostic Impact of Perioperative CA19-9 Levels in Patients with Resected Perihilar Cholangiocarcinoma

**DOI:** 10.3390/jcm10071345

**Published:** 2021-03-24

**Authors:** Jong Woo Lee, Jae Hoon Lee, Yejong Park, Jaewoo Kwon, Woohyung Lee, Ki Byung Song, Dae Wook Hwang, Song Cheol Kim

**Affiliations:** 1 Department of Surgery, Hallym University Sacred Heart Hospital, Anyang 14068, Korea; jongw.lee0212@gmail.com; 2 Department of Hepatobiliary and Pancreatic Surgery, Asan Medical Center, University of Ulsan College of Medicine, Seoul 05505, Korea; blackpig856@gmail.com (Y.P.); skunlvup@naver.com (J.K.); ywhnet@gmail.com (W.L.); mtsong21c@naver.com (K.B.S.); dwhwang@amc.seoul.kr (D.W.H.); drksc@amc.seoul.kr (S.C.K.)

**Keywords:** perihilar cholangiocarcinoma, prognosis, CA19-9, tumor marker

## Abstract

We aimed to examine the predictive value of changes in perioperative carbohydrate antigen (CA) 19-9 levels for patients operated for perihilar cholangiocarcinoma (pCCA). A total of 322 patients who underwent curative resection for pCCA were divided into three groups: normal preoperative CA19-9 (CA19-9 ≤ 37 U/mL), normalization (preoperative CA19-9 > 37 U/mL, postoperative CA19-9 ≤ 37 U/mL), and non-normalization (pre- and postoperative CA19-9 > 37 U/mL) groups. The association of clinicopathological factors with overall survival (OS) was investigated. The non-normalization group (*n* = 82) demonstrated significantly worse OS than the normal CA19-9 (*n* = 114) and normalization (*n* = 126) groups (5-year OS, 16.9%, 29.4%, and 34.4%, respectively; both *p* ≤ 0.001). The cutoff points of 300 U/mL for preoperative (*p* = 0.001) and 37 U/mL for postoperative (*p* < 0.001) CA19-9 levels showed the strongest prognostic values. In the non-normalization group, patients who underwent R1 resection displayed significantly worse OS than those who underwent R0 resection (median OS, 10.2 vs. 15.7 months; *p* = 0.016). Multivariate analysis revealed that lymph node metastasis (hazard ratio (HR), 2.07; *p* < 0.001), postoperative CA19-9 > 37 U/mL (HR, 1.94; *p* < 0.001), transfusion (HR, 1.74; *p* = 0.002), and T stage (T3,4) (HR, 1.67; *p* = 0.006) were related to worse OS. Persistent high CA19-9 level after resection of pCCA and R1 resection, especially in the non-normalization group, was associated with poor OS. A high postoperative CA19-9 level was an independent prognostic factor in resected pCCA.

## 1. Introduction

Perihilar cholangiocarcinoma (pCCA) is defined as tumor of the bile duct, involving or located within 2 cm of the first-order confluence of the common hepatic duct. It is classified as extrahepatic bile duct cancer with distal cholangiocarcinoma. pCCA is a rare disease with an annual incidence of one to two cases per 100,000 individuals, but is the fourth most common gastrointestinal malignancy [1]. Curative resection of pCCA is a demanding procedure and often exhibits poor prognosis, even after radical resection.

Numerous studies have investigated prognostic factors in resected pCCA. Several studies have reported serum tumor markers to have prognostic value. These markers are commonly used in clinical practice to assess and monitor the response to treatment in many malignancies. Among these, carbohydrate antigen 19-9 (CA19-9) is a tumor-associated antigen that was isolated from the human colorectal cancer cell line by Koprowski et al. in 1979 [2]. Since the 1980s, CA19-9 has been widely used to diagnose, predict prognosis, and monitor malignancies such as pancreatic and biliary tract cancers (BTC) [3]. Several studies have suggested that a decline in postoperative serumCA19-9 levels in pancreatic cancer is associated with improved survival of patients [4,5]. Regarding BTC, some studies have suggested that non-normalization of CA19-9 levels after resection was associated with worse prognosis [6], and elevated preoperative and postoperative CA19-9 levels were predictive of poor prognosis [7].

Several reports have suggested that elevated preoperative CA19-9 levels in patients with pCCA is associated with poor prognosis [8,9,10]. On the contrary, studies have reported that preoperative CA19-9 levels displayed no prognostic effect in patients with resected pCCA [11,12]. Therefore, the clinical significance of CA19-9 in pCCA patients as a prognostic marker remains unclear, demanding further investigation.

In this study, we examined the correlation between the change in perioperative CA19-9 levels and overall survival (OS) in patients with pCCA. We aimed to evaluate the predictive ability of CA19-9 for prognosis after surgical resection of pCCA.

## 2. Materials and Methods

### 2.1. Patients

We identified 425 patients who underwent curative surgery (including R0 and R1) for pCCA from 2008 to 2015 at the Department of Hepatobiliary and Pancreatic Surgery at Asan Medical Center, Seoul, Korea. The electronic medical records of these patients were retrospectively reviewed. pCCA was defined as the cholangiocarcinoma involving the hilum (the duct located between the right side of the umbilical portion of the left portal vein and the left side of the origin of the right posterior portal vein) based on a previous study [13]. Among them, we excluded 12 patients who underwent combined hepato-pancreaticoduodenal resection and 65 patients who underwent only extrahepatic bile duct resection. Of the remaining 348 patients who underwent major hepatectomy with extrahepatic bile duct resection, we included 322 patients with data on measured preoperative and postoperative serum CA19-9 levels in our final analysis.

For the initial evaluation, laboratory testing and an imaging workup were performed with dynamic computed tomography (CT) and magnetic resonance imaging (MRI) with magnetic resonance cholangiopancreatography (MRCP). An endoscopic biliary biopsy was performed on patients with suspicious cholangiocarcinoma for pathologic confirmation. Based on the images, biliary drainage (percutaneous or endoscopic) was also performed in patients with hyperbilirubinemia, obstructive cholangitis, or bile duct dilatation. For the detection of peritoneal and distant disease, a chest CT and positron emission tomography (PET)-CT were performed. In the patients who were determined to be resectable, total liver volume and future remnant liver volume was calculated, and an indocyanine green retention test (ICG R15) test was also examined. When future liver remnant volume was considered insufficient, we performed portal vein embolization.

Their preoperative serum CA19-9 levels were measured before the surgery. Since serum CA19-9 levels can be affected by hyperbilirubinemia, in the patients with jaundice at presentation, the CA19-9 level was measured after the total bilirubin level reached 3 mg/dL or below. As 268 patients (83.2%) exhibited obstructive jaundice or cholangitis at their first presentation, we performed preoperative biliary drainage—endoscopic retrograde or percutaneous transhepatic biliary drainage. The postoperative serum CA19-9 level was measured between 1 and 3 months. A normal serum CA19-9 level was defined as <37 U/mL.

The patients were divided into three groups: normal preoperative CA19-9 (CA19-9 ≤ 37 U/mL), normalization (preoperative CA19-9 >37 U/mL, postoperative CA19-9 ≤ 37 U/mL), and non-normalization (pre- and postoperative CA19-9 > 37 U/mL) groups. To find a significant cutoff point, we also categorized patients on the basis of preoperative and postoperative CA19-9 levels of 37, 100, 150, 200, 300, 400, and 1000 U/mL. These cutoff values of CA19-9 were based on the reference of previous studies [5,7,8,10,14].

Adjuvant chemotherapy was considered in case of R1 resection, lymph node metastasis, or stage II or above in the final pathology. The hepatobiliary oncologist decided whether to conduct chemotherapy in the outpatient 4–6 weeks after surgery. Gemcitabine and 5-fluorouracil with folic acid was mainly used for adjuvant chemotherapy. Adjuvant radiation therapy was also considered in the patient with R1 resection.

Patients were followed up on and monitored regularly in the outpatient department after discharge. Laboratory tests, including serumCA19-9 measurement and a computed tomography (CT) scan, were conducted at every visit. This retrospective study was approved by the institutional review board at Asan Medical Center (approval number: 2020-0130).

### 2.2. Preoperative Management

For the preoperative workup, we conducted CT and magnetic resonance cholangiopancreatography (MRCP) to determine the extent of the tumor, i.e., the involvement of a major vessel (portal vein and hepatic artery) or adjacent organ. Preoperative biliary drainage of the presumed remnant liver was performed using a percutaneous or endoscopic method when serum bilirubin was elevated, symptoms of cholangitis developed, or bile duct dilatation was visible in the image. Overall and future remnant liver volumes were measured based on preoperative CT scans. Portal vein embolization was performed to induce compensatory hypertrophy when the remnant liver volume did not meet our institutional criteria, and the liver volume was reevaluated three or four weeks later.

### 2.3. Surgical Procedures

In this study, all patients underwent major hepatectomy, including hemihepatectomy, trisectionectomy, and central bisectionectomy, accompanied with extrahepatic bile duct resection. All procedures included en-bloc resection of the specimen, including the caudate lobe. Regional lymph nodes—the hepatoduodenal ligament, pancreatoduodenal, and common hepatic artery lymph nodes—were routinely dissected in all patients. We routinely conducted frozen section examination of the proximal and distal bile duct resection margin intraoperatively.

### 2.4. Statistical Analysis

The patients were divided into three groups based on the change in their serum CA19-9 levels. Clinicopathological factors were compared among the groups using χ^2^ test and Fisher’s exact test. A paired samples *t*-test was employed to compare continuous variables. The Kaplan–Meier analysis and log-rank test were used to analyze the OS between groups divided by the serum CA19-9 levels and margin status. The prognostic value was considered to be the strongest in the most significant between-group difference (i.e., having the lowest *p*-value) in OS. Youden’s index was calculated to compare the prognostic value for variables with same *p*-values. The pre- and postoperative CA19-9 cutoff values (300 and 37 U/mL) showing the strongest prognostic ability were adopted in univariate and multivariate analyses for prognostic factors. Multivariate proportional hazards regression analysis was used to examine the impact of prognostic factors on OS. All statistical analyses were performed using SPSS Statistics for Windows version 25.0 (IBM Corp., Armonk, NY, USA).

## 3. Results

### 3.1. Patient Demographics and Clinical Characteristics

Table 1 summarizes the demographics and clinicopathological data of patients according to their CA19-9 grouping. Compared to normalization and non-normalization groups, the normal preoperative CA19-9 group exhibited a lower rate of jaundice at initial presentation (both *p* = 0.001). Compared to normal preoperative CA19-9 and normalization groups, the non-normalization group exhibited poorer OS (*p* = 0.003 and *p* < 0.001, respectively), shorter recurrence-free survival (RFS) (*p* = 0.006 and *p* = 0.004, respectively), and a higher rate of lymph node metastasis (*p* < 0.001 and *p* = 0.007, respectively). In terms of tumor marker levels, the non-normalization group showed a higher level of preoperative CA19-9 than the normalization group (*p* = 0.007). There was no significant difference in age, gender, body mass index (BMI), Bismuth type, hospital stay, 90-day mortality, or adjuvant treatment.

### 3.2. Prognostic Value of Pre- and Postoperative CA19-9 Levels

We calculated differences in OS divided by six cut-off values (37, 100, 200, 300, 400, and 1000 U/mL) of preoperative and postoperative CA19-9 levels (Table 2). The preoperative CA19-9 value of 300 U/mL demonstrated the strongest predictive value (*p* = 0.001), whereas the postoperative values of 37, 100, 200, and 300 U/mL showed high predictive values (*p* < 0.001). Based on Youden’s index, 37 was regarded as the postoperative cutoff value with the strongest prognostic value for survival. We performed multivariate analysis using the cutoff value of CA19-9 that exhibited the largest difference in OS between two groups (variable with the lowest *p*-value).

### 3.3. Outcomes According to Perioperative CA19-9 Status

**Figure 1:**
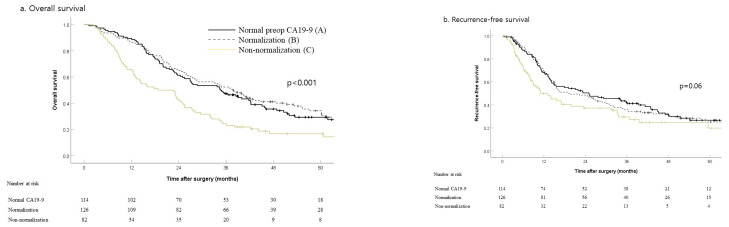
Long-term outcomes by perioperative carbohydrate 19-9 (CA19-9) status in 322 patients who underwent resection with curative intent of perihilar cholangiocarcinoma (A: preoperative normal CA19-9, B: high preoperative and normalized postoperative CA19-9, and C: high preoperative and non-normalized postoperative CA19-9): (**a**) overall survival, *p* = 0.475 (A vs. B), *p* = 0.001 (A vs. C), *p* < 0.001 (B vs. C) and (**b**) recurrence-free survival, *p* = 0.854 (A vs. B), *p* = 0.046 (A vs. C), *p* = 0.077 (B vs. C).

The five-year OS rate was worse in the non-normalization group (16.9%) than in the normal (29.4%, *p* = 0.001) and normalization (34.4%, *p* < 0.001) groups (Figure 1a). The three-year RFS rate of the non-normalization group (29.5%) was significantly poorer than the normal group (42.4%, *p* = 0.046) but showed marginal difference with the normalization group (36%, *p* = 0.077) (Figure 1b).

**Figure 2:**
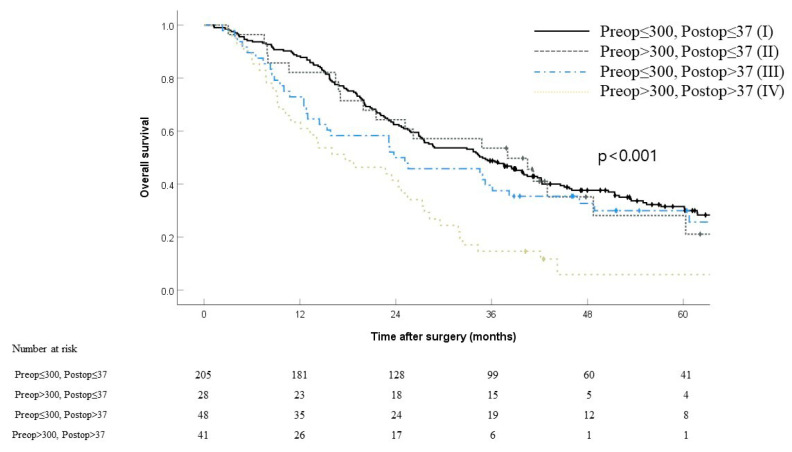
Kaplan–Meier curves comparing overall survival stratified by preoperative and postoperative CA19-9 levels (I: preop ≤ 300, postop ≤ 37, II: preop > 300, postop ≤ 37, III: preop ≤ 300, postop > 37, IV: preop > 300, postop > 37). *p* = 0.720 (I vs. II), *p* = 0.205 (I vs. III), *p* < 0.001(I vs. IV), *p* = 0.643 (II vs. III), *p* = 0.003 (II vs. IV), *p* = 0.009 (III vs. IV).

Patients were divided into four groups according to their preoperative (300 U/mL) and postoperative (37 U/mL) CA19-9 cutoff levels—which showed the strongest prognostic impacts. We conducted a comparative analysis of survival among the groups (Figure 2). The four groups comprised low preoperative/low postoperative CA19-9 (*n* = 205, 63.7%), high preoperative/low postoperative CA19-9 (*n* = 28, 8.7%), low preoperative/high postoperative CA19-9 (*n* = 48, 14.9%), and high preoperative/high postoperative CA19-9 (*n* = 41, 12.7%). The median OS of each group was 34.9, 38.0, 23.8, and 17.7 months, respectively. Patients with a high preoperative/high postoperative CA19-9 had a five-year OS that was considerably poorer (5.9%) than those with low preoperative/low postoperative CA19-9 (31.5%, *p* < 0.001), high preoperative/low postoperative CA19-9 (28.1%, *p* = 0.003), or low preoperative/high postoperative CA19-9 (30.0%, *p* = 0.009). Based on the logistic regression analysis of factors associated with one-year mortality, the tumor group based on preoperative and postoperative CA19-9 remained strongly associated with one-year mortality (Supplementary Table S1). Specifically, patients with low preop/high postop CA19-9 (OR 4.76; 95% CI 1.42-16.02; *p* = 0.012) and high preop/high postop CA19-9 (OR 6.62; 95% CI 2.10-20.83; *p* = 0.001) had higher odds of one-year mortality compared to patients with low preop/low postop CA19-9, respectively. On the other hand, the high preop/low postop CA19-9 group (OR 2.49; 95% CI 0.62-9.97; *p* = 0.199) did not increase one-year mortality significantly compared to the low preop/low postop CA19-9 group.

### 3.4. Effect of Changes in Perioperative CA19-9 Levels on Survival

**Figure 3:**
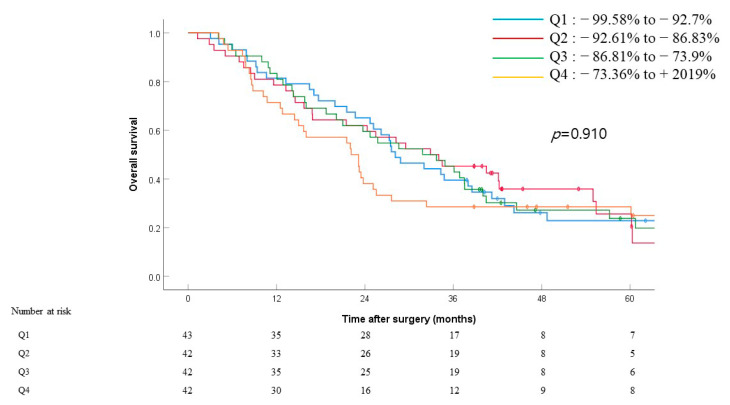
Overall survival by ΔCA19-9 values after surgery among patients with preop CA19-9 >60 U/mL (*n* = 169). Patients are categorized by quartiles of ΔCA19-9, with Q1 representing the greatest decline in CA19-9 and Q4 representing the least decline in CA19-9 or increase in CA19-9 after surgery (ΔCA19-9 = (postop CA19-9 − preop CA19-9)/preop CA19-9). *p* = 0.838 (Q1 vs. Q2), *p* = 0.814 (Q1 vs. Q3), *p* = 0.750 (Q1 vs. Q4), *p* = 0.856 (Q2 vs. Q3), *p* = 0.517 (Q2 vs. Q4), *p* = 0.485 (Q3 vs. Q4).

To assess the effect of the rate of the change in levels in this tumor marker, we calculated this change in patients with preoperative CA19-9 levels > 60 U/mL (*n* = 169) (Figure 3) using the following formula:

ΔCA19-9 = (postop CA19-9 − preop CA19-9)/preop CA19-9

We then categorized patients into four quartiles (Q1–4) based on their ΔCA19-9 values (Figure 3), with Q1 consisting of patients with the greatest ΔCA19-9 and Q4 consisting of patients with the smallest ΔCA19-9. The median OS for patients in Q1–4 were 28.1, 32.9, 31.8, and 22.1 months, respectively. There was no significant difference in survival between each group (Q1 vs. Q2, *p* = 0.838; Q1 vs. Q3, *p* = 0.814; Q1 vs. Q4, *p* = 0.750; Q2 vs. Q3, *p* = 0.856; Q2 vs. Q4, *p* = 0.517; Q3 vs. Q4, *p* = 0.485). The rate of change in CA19-9 did not show a significant impact on OS (*p* = 0.910) (Figure 3).

### 3.5. Univariate and Multivariate OS Analyses for Clinicopathological Factors

We performed univariate and multivariate analyses to investigate the prognostic factors associated with OS (Table 3). The multivariate analysis identified pathologic stages (III and IV) (hazard ratio (HR), 2.35; 95% confidence interval (CI), 1.59–3.48; *p* < 0.001), transfusion (HR, 1.85; 95% CI, 1.30–2.64; *p* = 0.001), and postoperative CA19-9 level (>37 U/mL) (HR, 1.80; 95% CI, 1.27–2.55; *p* = 0.001) as independent predictors of poor OS. The quartile of ΔCA19-9 and the preoperative CA19-9 level (>300 U/mL) did not display significant predictability of OS in the multivariate analysis.

### 3.6. Outcomes According to Resection Margin in Patients with Elevated Preoperative CA19-9

**Figure 4:**
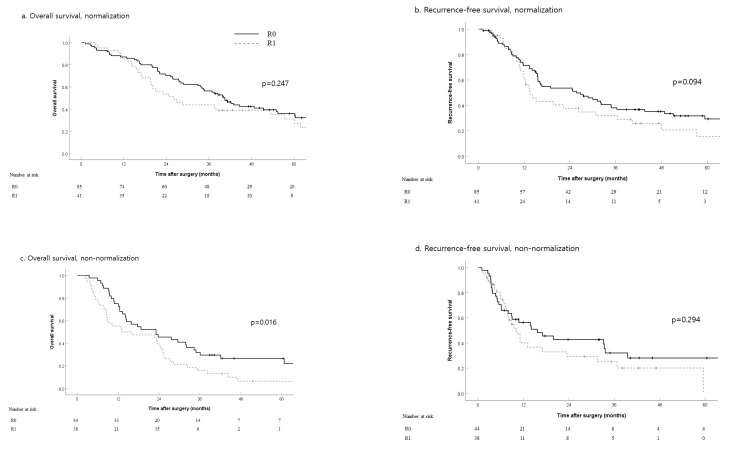
Overall survival and recurrence-free survival by resection margin status in patients with elevated CA19-9 level that normalized (**a**,**b**) and did not (**c**,**d**) normalize after surgery. (**a**) Overall survival, normalization; (**b**) recurrence-free survival, normalization; (**c**) overall survival, non-normalization; (**d**) recurrence-free survival, non-normalization.

Among patients whose postoperative CA19-9 levels normalized, resection margins R0 and R1 did not significantly affect their five-year OS (36% vs. 31% respectively, *p* = 0.247) or three-year RFS (38% vs. 29%, *p* = 0.094) (Figure 4a,b). In the non-normalized group, patients who underwent R1 resection had worse OS than those with R0 resection (5-year OS, 26% vs. 6%; *p* = 0.016) (Figure 4c,d).

## 4. Discussion

This study demonstrated that a sustained increase in CA19-9 level after resection of pCCA was an indicator of poor prognosis. On the contrary, patients with high preoperative CA19-9 levels that normalized after surgery exhibited an OS similar to that of patients with normal preoperative CA19-9 levels. Additionally, among patients with persistent high CA19-9 levels after surgery, those who underwent R1 resection displayed significantly worse OS than those who underwent R0 resection. In contrast, patients with postoperative normalized CA19-9 levels displayed equivalent OS and RFS, irrespective of R1 or R0 resection. The analysis of the impact of changes in serum CA19-9 levels on survival revealed no significant difference among the groups divided by decrease rate.

As mentioned in the Methods section, surgery was considered if there was no evidence of distant metastasis in patients with even an extensive high CA19-9 level. In this study, 25 patients with preoperative CA19-9 of more than 1000 U/m, which might reflect high tumor burden, were included. A total of 14 patients survived for more than 24 months and 3 patients survived for more than 60 months after surgery. For postoperative CA19-9, serum CA19-9 was normalized in 4 patients and declined less than 100 U/mL in 11 patients. We actively performed curative resection if the patient was considered resectable in consideration of major vessel invasion, lymph node metastasis, tumor extent, and liver volume.

This is the first study that evaluated the prognostic impact of not only multiple cutoff values of both pre- and postoperative CA19-9 levels, but also perioperative changes in more than 100 patients with pCCA in a single institution. Several previous studies reported the prognostic value of perioperative CA19-9 levels in patients undergoing resection for BTC [7,15,16,17] as well as pancreatic cancer [5,18,19]. BTC, including intrahepatic cholangiocarcinoma, pCCA, and distal cholangiocarcinoma, has often been investigated for statistical power because of its rare incidence with each disease [7]. Several studies reported elevated preoperative [16,17] and postoperative [7,15] CA19-9 levels as independent prognostic factors for worse OS in resected BTC. To the best of our knowledge, only one previous study analyzed the prognostic effect of pre- and postoperative serum CA19-9 levels on survival in 98 patients with resected pCCA [14].

Unlike previous studies [16,17], our study did not prove that preoperative CA19-9 level is an independent prognostic factor for pCCA. In our study, patients with high preoperative CA19-9 levels that normalized after surgery displayed an OS similar to that of patients with preoperative normal CA19-9 levels. This supports that a change in the perioperative serum CA19-9 level is more crucial to the long-term outcome than the absolute preoperative level. Figure 2 illustrates that among patients with a preoperative CA19-9 level >300 U/mL, those with a normalized level (<37 U/mL) after surgery (5-year OS, 28.1%) exhibited a significantly better OS than those with a non-normalized level (>37 U/mL) (5-year OS, 28.1%; *p* = 0.003). Patients with elevated preoperative (>300 U/mL) and postoperative (>37 U/mL) serum CA19-9 levels showed significantly worse OS than any other groups. Based on these results, the preoperative CA19-9 level alone is insufficient for predicting prognosis before the surgery. In this study, we categorized patients according to various cutoff values of both pre- and postoperative serum CA19-9 levels—37, 100, 200, 300, 400, and 1000 U/mL—adopted from several previous studies [5,7,14]. Of these cutoff values, 300 U/mL for preoperative (*p* = 0.001) and 37, 100, 200, and 300 U/mL for postoperative (*p* < 0.001) CA19-9 levels showed significant difference in the patients’ OS. Through further analysis, we observed that 300 U/mL was the strongest prognostic cutoff value for postoperative CA19-9 levels. To our knowledge, only one study [14] has analyzed the association between postoperative CA19-9 level and survival in pCCA.

We also investigated the rate of change in CA19-9 levels to observe its impact on OS. We categorized patients into four quartiles according to their ΔCA19-9 and analyzed the survival between each quartile group. Although there was no significant difference in OS between any quartile groups, we would need to further evaluate the magnitude of change in the perioperative serum CA19-9 level.

In multivariate analysis, we observed that lymph node metastasis (HR, 2.07; *p* < 0.001), increased postoperative CA19-9 levels (HR, 1.94; *p* < 0.001), transfusion (HR, 1.74; *p* = 0.002), and advanced T stage (HR, 1.67; *p* = 0.006) were independent prognostic factors. On the contrary, margin status was not a significant prognostic factor in multivariate analysis, inconsistent with the previous studies [8,13,20].

Increased preoperative CA19-9 levels that did not normalize after surgery (non-normalization) was a poor prognostic factor; it would be reasonable to consider adjuvant therapy in patients belonging to this group, especially those who underwent R1 resection (Figure 2). In addition, patients with normalized CA19-9 levels after surgery exhibited OS and RFS comparable to those who underwent R0 resection; therefore, we can expect comparable survival even though patients underwent R1 resection (Figure 4a,b). Despite the results on margin status, achieving R0 resection should not be overlooked because a clear resection margin is considered one of the most important prognostic factors, based on a previous study [21].

In this study cohort, we did not exclude or adjust for patients with preoperative hyperbilirubinemia (total bilirubin level > 2 mg/dL) for the following reasons. First, excluding patients with hyperbilirubinemia could cause selection bias. Second, it is still unclear whether it is appropriate to calculate adjusted CA19-9 values for patients with hyperbilirubinemia. Third, a previous study reported that serum CA19-9 levels are not significantly affected by hyperbilirubinemia in patients with pancreatic cancer and chronic pancreatitis [22], and that it is not essential to use adjusted CA19-9 values in such patients. Nevertheless, to minimize the possible impact, we measured the preoperative CA19-9 level after the jaundice improved.

In Korean patients with gastric cancer, approximately 10.5% (6/57 patients) have been reported to be of the homozygous Lewis genotype (le/le), which has neither the Le^a^ nor the Le^b^ antigen [23]. Based on the above reference, 11 patients (10.5% of 114) might be Lewis antigen-negative in the normal CA19-9 group (Table 1). Considering these false-negative patients, the maximum sensitivity of CA19-9 would be approximately 90% and should be considered when interpreting the results. Indeed, there are several prior studies [18,24,25,26] that supported that Lewis antigen-negative (Le(a-b-)) individuals cannot produce CA19-9. On the other hand, there are some other studies [27,28,29] that supported that individuals with Le(a-b-) may still produce CA19-9 and that their serum concentration of CA19-9 is clinically relevant. It is difficult to draw a clear conclusion about Lewis antigen phenotypes and CA19-9 secretion yet. Although we could not obtain information about Lewis antigen phenotypes in this cohort, further research considering the Lewis antigen could contribute to producing a clear conclusion on this matter.

There are some limitations of this study because of its retrospective and non-randomized nature. To clarify the prognostic impact of CA19-9 levels on pCCA, we require future prospective analyses with a larger study population and more clinical variables. We believe that these studies would enable us to stratify patients according to their prognoses and contribute to the optimization of perioperative treatment strategies in pCCA.

## 5. Conclusions

In conclusion, the present study demonstrated that persistent high serum CA19-9 levels after curative resection indicates poor prognosis in patients with pCCA. Perioperative serum CA19-9 level and margin status can help in strategizing the adjuvant treatment. Moreover, an elevated postoperative serum CA19-9 level is an independent factor that predicts poor prognosis in resected pCCA.

## Figures and Tables

**Table 1:** Clinicopathological characteristics stratified by CA19-9 status of patients who underwent curative resection of perihilar cholangiocarcinoma (*n* = 322).

	Normal CA19-9 (A, *n* = 114)	Normalization (B, *n* = 126)	Non-Normalization (C, *n* = 82)	*p*-Value		
				A vs. B	A vs. C	B vs. C
Age (years)	64.2 ± 9.0	63.1 ± 9.6	63.6 ± 8.0	0.364	0.617	0.714
Sex ratio (male:female)	76:38	89:37	52:30	0.508	0.637	0.276
BMI (kg/m^2^)	23.6 ± 3.0	23.0 ± 2.7	22.8 ± 2.6	0.129	0.068	0.595
Jaundice at presentation	47 (41.2%)	80 (63.5%)	54 (65.9%)	0.001	0.001	0.728
Albumin (g/dL)	3.4 ± 0.4	3.3 ± 0.5	3.3 ± 0.4	0.088	0.026	0.541
Total bilirubin (mg/dL)	1.5 ± 1.2	1.9 ± 1.4	2.1 ± 1.5	0.053	0.011	0.364
Preoperative biliary drainage				0.025	0.124	0.723
None	25 (21.9%)	18 (14.3%)	11 (13.4%)			
Percutaneous transhepatic drainage	54 (47.4%)	80 (63.5%)	47 (57.3%)			
Endoscopic drainage	31 (27.2%)	20 (15.9%)	17 (20.7%)			
Both	4 (3.5%)	8 (6.3%)	7 (8.5%)			
Preoperative cholangitis	20 (17.5%)	24 (19.0%)	23 (28.0%)	0.764	0.080	0.129
Preoperative CA19-9 (U/mL)	16.3 ± 9.9	245.4 ± 441.9	1420.3 ± 3860.6	<0.001	0.001	0.007
Portal vein embolization	2 (1.8%)	2 (1.6%)	4 (4.9%)	1.000	0.239	0.215
Liver resection type				0.130	0.040	0.136
RH and ERH	77 (67.5%)	73 (57.9%)	55 (67.1%)			
RTS	2 (1.8%)	6 (4.8%)	5 (6.1%)			
LH and ELH	32 (28.1%)	37 (29.4%)	14 (17.1%)			
LTS	3 (2.6%)	10 (7.9%)	6 (7.3%)			
CBS	0	0	2 (0.6%)			
Portal vein resection	12 (10.5%)	22 (17.5%)	16 (19.5%)	0.124	0.076	0.708
R1 resection	42 (36.8%)	41 (32.5%)	38 (46.3%)	0.484	0.182	0.045
Intraoperative transfusion	38 (33.3%)	47 (37.3%)	31 (37.8%)	0.521	0.518	0.942
Operative time(min)	357.9 ± 75.0	362.5 ± 69.4	380.5 ± 78.8	0.625	0.043	0.084
Bismuth type				0.507	0.149	0.800
I	5 (4.4%)	5 (4.0%)	2 (2.4%)			
II	22 (19.3%)	18 (14.3%)	8 (9.8%)			
IIIa	50 (43.9%)	57 (45.2%)	37 (45.1%)			
IIIb	22 (19.3%)	20 (15.9%)	15 (18.3%)			
IV	15 (13.2%)	26 (20.6%)	20 (24.4%)			
TNM stage (AJCC 8th)				0.226	<0.001	0.063
I	19 (16.7%)	10 (7.9%)	4 (4.9%)			
II	48 (42.1%)	48 (38.1%)	18 (22.0%)			
IIIA	10 (8.8%)	14 (11.1%)	7 (8.5%)			
IIIB	3 (2.6%)	6 (4.8%)	6 (7.3%)			
IIIC	28 (24.6%)	36 (28.6%)	29 (35.4%)			
IVA	6 (5.3%)	9 (7.1%)	14 (17.1%)			
IVB	0	3 (2.4%)	4 (4.9%)			
Hospital stay (days)	20.7 ± 12.6	20.0 ± 12.2	23.9 ± 15.2	0.659	0.104	0.050
90-day mortality	1 (0.9%)	0	1 (1.2%)	0.475	1.000	0.394
Postoperative CA19-9 (U/mL)	14.7 ± 17.2	20.0 ± 9.3	186.1 ± 349.2	0.003	<0.001	<0.001
Overall survival (months)	36.5 ± 24.3	39.7 ± 26.7	25.9 ± 23.2	0.334	0.003	0.000
Recurrence-free survival (months)	28.0 ± 23.9	28.6 ± 26.6	18.6 ± 22.1	0.862	0.006	0.004
Adjuvant chemotherapy	44 (38.6%)	52 (41.3%)	33 (40.2%)	0.673	0.816	0.883
Adjuvant radiotherapy	25 (21.9%)	23 (18.3%)	19 (23.2%)	0.477	0.837	0.388
Tumor size (cm)	2.9 ± 1.5	3.3 ± 1.5	3.5 ± 1.9	0.054	0.015	0.451
LN metastasis	33 (28.9%)	48 (38.1%)	47 (57.3%)	0.134	<0.001	0.007
Perineural invasion	88 (77.2%)	110 (87.3%)	71 (86.6%)	0.040	0.097	0.881
Lympho-vascular invasion	55 (48.2%)	57 (45.2%)	48 (58.5%)	0.641	0.155	0.061
Recurrence	75	84	53	0.901	0.048	0.070
Locoregional	30 (26.3%)	32 (25.4%)	9 (11.0%)			
Systemic	29 (25.4%)	37 (29.4%)	30 (36.6%)			
Both	16 (14.0%)	15 (11.9%)	14 (17.1%)			

CA 19-9, carbohydrate antigen 19-9; BMI, body mass index; RH, right hemihepatectomy; ERH, extended right hemihepatectomy; RTS, right trisectionectomy; LH, left hemihepatectomy; ELH, extended left hemihepatectomy; LTS, left trisectionectomy; CBS, central bisectionectomy; TNM, tumor, node, metastasis; AJCC, American Joint Committee on Cancer; LN, lymph node.

**Table 2:** Difference of overall survival in two groups divided on the basis of CA19-9 cutoff values.

CA19-9 Cut-off Value (U/mL)	Preoperative CA19-9			Postoperative CA19-9		
	Patient Number (n)	Median Survival (Months)	*p*-Value	Patient Number (n)	Median Survival (Months)	*p*-Value
≤37	114	34.53	0.300	233	35.23	0.000
>37	208	27.47		89	23.13	
≤100	190	33.70	0.138	288	34.37	0.000
>100	132	27.30		34	10.87	
≤200	229	33.70	0.027	307	33.70	0.000
>200	93	26.20		15	8.50	
≤300	253	34.37	0.001	309	32.90	0.000
>300	69	24.3		13	8.50	
≤400	270	33.70	0.003	315	29.53	0.003
>400	52	24.30		7	12.07	
≤1000	297	32.90	0.007	318	28.83	0.001
>1000	25	24.70		4	5.40	

**Table 3:** Univariate and multivariate analyses of predictors of overall survival after surgery for 322 resected patients.

	Univariate			Multivariate		
	HR	95% CI	*p*-Value	HR	95% CI	*p*-Value
Age (years)	1.01	0.99–1.02	0.390			
Albumin (g/dL)	0.92	0.70–1.20	0.528			
Total bilirubin (mg/dL)	1.07	0.98–1.17	0.111			
Prothrombin time (min)	1.39	0.38–5.00	0.618			
Quartile of ΔCA19-9			0.425			
Q1	*Ref.*					
Q2	0.90	0.58–1.40	0.636			
Q3	0.88	0.57–1.37	0.578			
Q4	1.23	0.80–1.88	0.350			
Preop CA19-9 >300 U/mL	1.62	1.20–2.19	0.001			
Postop CA19-9 >37 U/mL	1.64	1.25–2.16	<0.001	1.94	1.36–2.77	<0.001
CA19-9 status			<0.001			
Normal CA19-9	*Ref.*					
Normalization	0.90	0.67–1.22	0.512			
Non-normalization	1.75	1.27–2.40	0.001			
Liver resection type			0.312			
RH or ERH	*Ref.*					
LH or ELH	0.90	0.67–1.21	0.478			
LTS	1.11	0.64–1.92	0.708			
RTS	1.21	0.66–2.24	0.539			
CBS	3.78	0.93–15.33	0.063			
Portal vein resection	1.52	1.09–2.12	0.014			
Intraoperative transfusion	1.51	1.17–1.96	0.002	1.74	1.22–2.48	0.002
R1 resection	1.02	0.90–1.15	0.756			
Size (cm)	1.07	0.99–1.15	0.089			
Poorly differentiated	1.27	0.86–1.87	0.226			
Portal vein invasion	1.88	1.35–2.61	<0.001			
Lymphovascular invasion	1.71	1.33–2.21	<0.001			
Perineural invasion	1.43	1.01–2.04	0.046			
LN metastasis	2.02	1.56–2.60	<0.001	2.07	1.45–2.97	<0.001
T stage (T3,4 vs. T1,2)	1.83	1.40–2.40	<0.001	1.67	1.16–2.41	0.006
Adjuvant chemotherapy	1.36	1.06–1.76	0.016			
Adjuvant radiotherapy	1.42	1.06–1.90	0.019			

HR, hazard ratio; CI, confidence interval.

## Data Availability

Data sharing not applicable.

## Supplementary Materials

The following are available online at https://www.mdpi.com/2077-0383/10/7/1345/s1, Table S1: Logistic regression analysis of perioperative prognostic factors associated with 1-year mortality.
