# Supplementary material for: Prognostic Impact of Perioperative CA19-9 Levels in Patients with Resected Perihilar Cholangiocarcinoma
Source: J Clin Med. 2021 Mar 24;10(7):1345. doi: 10.3390/jcm10071345 (PMC8036534; doi:10.3390/jcm10071345)
Supplement: Supplementary file 1 [file jcm-10-01345-s001.pdf]

Supplementary Table S1. Logistic regression analysis of perioperative prognostic factors associated with 1-year mortality.

	Univariate			Multivariate		
	**OR**	**95% CI**	***p*-value**	**OR**	**95% CI**	***p*-value**
Age (years)	1.01	0.98-1.05	0.450			
Albumin (g/dL)	0.90	0.47-1.71	0.742			
Total bilirubin (mg/dL)	1.13	0.93-1.38	0.214			
Prothrombin time (min)	13.71	0.84-223.98	0.066			
Quartile of ΔCA19-9			0.688			
Q1	*Ref*					
Q2	1.16	0.40-3.37	0.786			
Q3	1.00	0.34-2.97	1.000			
Q4	1.70	0.61-4.72	0.308			
Tumor marker group			**<0.001**			**0.009**
Low preop / Low postop CA19-9	*Ref*			*Ref*		
High preop / Low postop CA19-9	1.71	0.59-4.95	0.319	2.49	0.62-9.97	0.199
Low preop / High postop CA19-9	2.80	1.30-6.03	**0.008**	4.76	1.42-16.02	**0.012**
High preop / High postop CA19-9	4.83	2.26-10.30	**<0.001**	6.62	2.10-20.83	**0.001**
CA19-9 status						
Normal preoperative CA19-9	*Ref*					
Normalization	1.14	0.52-2.49	0.741			
Non-normalization	4.25	2.04-8.86	**<0.001**			
Portal vein resection	2.27	1.14-4.52	**0.019**			
Intraoperative transfusion	2.06	1.16-3.67	**0.014**	2.28	0.98-5.30	0.057
R1 resection	1.90	1.07-3.36	**0.030**			
Size (cm)	1.12	0.95-1.31	0.177			
Portal vein invasion	2.35	1.18-4.68	**0.015**			
Lymphovascular invasion	2.45	1.34-4.46	**0.003**			
Perineural invasion	1.89	0.77-4.65	0.168			
LN metastasis	3.39	1.87-6.13	**<0.001**	3.58	1.46-8.78	**0.005**
T stage (T3,4 vs. T1,2)	3.31	1.84-5.97	**<0.001**	3.08	1.30-7.30	**0.011**
Adjuvant chemotherapy	0.57	0.31-1.05	0.072	0.42	0.17-1.05	0.062
Adjuvant radiotherapy	0.77	0.37-1.62	0.490			
